# Supplementary material for: The Association of Beta-Blocker Use and Bone Mineral Density Level in Hemodialysis Patients: A Cross-Sectional Study
Source: Medicina (Kaunas). 2023 Jan 9;59(1):129. doi: 10.3390/medicina59010129 (PMC9860648; doi:10.3390/medicina59010129)
Supplement: Supplementary file 1 [file medicina-59-00129-s001.zip › medicina-2125750-supplementary.pdf]

**Table S1: Baseline characteristics of beta-1 selective blockers versus non-selective beta-blocker users**

| Characteristics | Beta-1 selective blockers (N=23) | Non-selective beta-blocker users (N=48) | *p*-Value |
|---|---|---|---|
| Age, years, mean (SD) | 66.6 (11.0) | 62.7 (17.5) | 0.34 |
| Female, *n* (%) | 13 (56.5) | 29 (60.4) | 0.80 |
| BMI, kg/m$^2$, mean (SD) | 23.6 (4.6) | 25.0 (5.3) | 0.26 |
| Alcohol, *n* (%) | | | 0.76 |
| • Never | 18 (78.3) | 33 (68.8) | |
| • Current | 1 (4.3) | 4 (8.3) | |
| • Former | 4 (17.4) | 11 (22.9) | |
| Former Smoking, *n* (%) | 4 (17.4) | 6 (12.5) | 0.72 |
| Underlying disease, *n* (%) | | | |
| • Dyslipidemia | 12 (52.2) | 28 (58.3) | 0.80 |
| • Ischemic stroke | 0 (0) | 6 (12.5) | 0.17 |
| • Ischemic heart disease | 7 (30.4) | 10 (20.8) | 0.40 |
| • Gout | 3 (13.0) | 7 (14.6) | 1.00 |
| • Hypertension | 23 (100.0) | 43 (89.6) | 0.17 |
| • Diabetes mellitus | 8 (34.8) | 27 (56.2) | 0.13 |
| • Atrial fibrillation | 2 (8.7) | 2 (10.4) | 1.00 |
| Dialysis vintage, months, median (IQR) | 38.0 (22.0-67.0) | 28.5 (16.0-64.5) | 0.28 |
| Antihypertensive drugs, *n* (%) | | | |
| • ACEi | 1 (4.3) | 2 (4.2) | 1.00 |
| • ARB | 8 (34.8) | 21 (43.8) | 0.61 |
| • Alpha - 1 blocker | 5 (21.7) | 14 (29.2) | 0.58 |
| • CCB | 18 (78.3) | 35 (72.9) | 0.77 |
| • Vasodilator | 9 (39.1) | 12 (25.0) | 0.27 |
| Statin, *n* (%) | 16 (69.3) | 32 (66.7) | 1.00 |
| Non-calcium-based phosphate binder, *n* (%) | 3 (13.0) | 7 (14.6) | 1.00 |
| Active vitamin D, *n* (%) | 13 (56.5) | 20 (41.7) | 0.31 |
| Laboratory | | | |
| • Calcium, mg/dL, median (IQR) | 9.0 (8.25-9.46) | 9.0 (8.5-9.4) | 0.89 |
| • Phosphate, mg/dL, median (IQR) | 4.7 (3.85-5.6) | 4.5 (3.85-5.17) | 0.64 |
| • PTH, pg/mL, median (IQR) | 457.0 (327.0-782.4) | 457.9 (300.9-624.0) | 0.66 |
| • PTH level, *n* (%) | | | 0.054 |
| PTH < 120 pg/mL | 2 (8.7) | 0 (0) | |
| PTH 120-585 pg/mL | 12 (52.2) | 37 (70.8) | |
| PTH >585 pg/mL | 9 (39.1) | 14 (29.2) | |
| • ALP, U/L, median (IQR) | 96.0 (69.0-154.0) | 82 (65.7-113.5) | 0.14 |
| • ALP level, *n* (%) | | | 1.00 |
| ALP ≤ 120 U/L | 40(70.18) | 50(70.42) | |
| ALP > 120 U/L | 17(29.82) | 21(29.58) | |
| • Vitamin D, ng/mL, median (IQR) | 41.8 (30.1-51.2) | 34.8 (24.8-48.4) | 0.25 |

| | | | |
|---|---|---|---|
| • Bicarbonate, mEq/L, median (IQR) | 24.0 (23.3-24.7) | 25.0 (23.8-26.5) | 0.03 |
| • nPCR, g/kg/day, median (IQR) | 1.1 (1.0-1.2) | 1.1 (0.9-1.3) | 0.90 |
| • URR, %, median (IQR) | 80.7 (79.0-84.0) | 80.2 (78.0-83.6) | 0.45 |
| • Albumin, g/dL, median (IQR) | 3.6 (3.4-3.8) | 3.7 (3.5-3.9) | 0.34 |
| Bone turnover*, *n* (%) | | | 0.18 |
| • High turnover | 4 (17.4) | 8 (16.7) | |
| • Low turnover | 2 (8.7) | 0 (0) | |
| • Undetermined | 17 (73.9) | 40 (83.3) | |

Abbreviation: ACEi, angiotensin-converting enzyme inhibitor; ALP, alkaline phosphatase; ARB, angiotensin II receptor blocker; BMI, body mass index; CCB, calcium channel blockers; nPCR, normalized protein catabolic rate; PTH, parathyroid hormone; URR, urea reduction ratio

*high turnover defined as high PTH and ALP, low turnover defined as low PTH and ALP, undetermined defined as high PTH with low ALP or low PTH with high ALP

**Table S2: Comparison of BMD and incidence of osteoporosis between beta-blocker and control group**

| BMD | Total (N=128) | Control group (N=57) | Beta-blocker group (N=71) | *p*-Value (Control VS BB) |
|---|---|---|---|---|
| **Total Lumbar spine** | | | | |
| BMD, median [IQR] | 0.92 [0.78-1.08] | 0.93 [0.79-1.05] | 0.91 [0.77-1.10] | 0.88 |
| T-score, median [IQR] | -0.85 [-2.00 to 0.55] | -0.7 [-1.90 to 0.30] | -0.90 [-2.10 to 0.80] | 0.91 |
| Osteoporosis, *n* (%) | 21 (16.4) | 9 (15.8) | 12 (16.9) | 1.00 |
| **Femoral neck** | | | | |
| BMD, median [IQR] | 0.59 [0.49-0.70] | 0.59 [0.50-0.70] | 0.57 [0.46-0.67] | 0.21 |
| T-score, median [IQR] | -2.05 [-2.90 to -1.10] | -2.0 [-2.80 to -1.00] | -2.20 [-3.00 to -1.20] | 0.39 |
| Osteoporosis, *n* (%) | 46 (35.94) | 18 (31.6) | 26 (36.6) | 0.58 |
| **Total hip** | | | | |
| BMD, median [IQR] | 0.71 [0.60-0.84] | 0.73 [0.61-0.85] | 0.70 [0.58-0.83] | 0.38 |
| T-score, median [IQR] | -1.40 [-2.20 to -0.30] | -1.40 [-2.10 to -0.50] | -1.50 [-2.30 to -0.20] | 0.59 |
| Osteoporosis, *n* (%) | 23 (17.97) | 9 (15.8) | 14 (19.7) | 0.65 |
| **1/3 Radius** | | | | |
| BMD, median [IQR] | 0.65[0.51-0.71] | 0.68[0.51-0.72] | 0.64[0.52-0.71] | 0.40 |
| T-score, median [IQR] | -2.00 [-2.95 to -0.75] | -2.00 [-2.90 to -0.70] | -2.1 [-3.10 to -0.80] | 0.74 |
| Osteoporosis, *n* (%) | 45 (35.16) | 19(33.33) | 26(36.62) | 0.71 |
| **Osteoporosis** | | | | |
| Osteoporosis any, *n* (%) | 64 (50.00) | 27 (47.37) | 37 (52.11) | 0.72 |

BB, beta-blocker users; BMD, bone mineral density

**Table S3: Association of BMD with beta-blocker subtypes**

| Site of BMD | Multivariate analyses | | | |
|---|---|---|---|---|
| | Beta-1 selective blocker | | Non-selective beta-blocker | |
| | Model A Coefficient (95%CI) *p*-Value | Model B Coefficient (95%CI) *p*-Value | Model A Coefficient (95%CI) *p*-Value | Model B Coefficient (95%CI) *p*-Value |
| **Total Spine** | -0.04 (-0.79 to 0.71) 0.92 | 0.01 (-0.75 to 0.75) 0.997 | 0.06 (-0.54 to 0.66) 0.84 | 0.08 (-0.53 to 0.69) 0.80 |
| **Femoral Neck** | -0.17 (-0.33 to -0.005) 0.04 | -0.16 (-0.33 to 0.003) 0.054 | -0.01 (-0.014 to 0.12) 0.83 | -0.01 (-0.15 to 0.12) 0.83 |
| **Total Hip** | -0.003 (-0.10 to 0.10) 0.95 | -0.003 (-0.10 to 0.10) 0.95 | 0.0009 (-0.08 to 0.08) 0.98 | 0.001 (-0.08 to 0.8) 0.98 |
| **1/3 Radius** | 0.009 (-0.04 to 0.06) 0.72 | 0.01 (-0.04 to 0.06) 0.65 | 0.02 (-0.04 to 0.04) 0.92 | 0.002 (-0.04 to 0.04) 0.92 |

BMD, bone mineral density

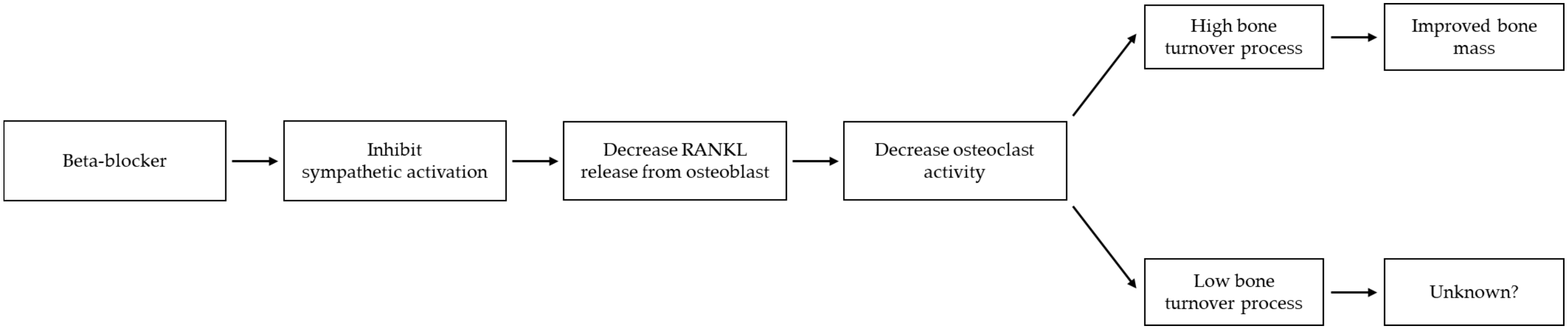


**Figure S1: Potential mechanism of beta-blocker use and bone remodeling in hemodialysis patients.**
Beta-blocker decreases bone remodeling via inhibition of sympathetic activation and decrease RANKL releasing from osteoblast which results in decreasing osteoclast activity. This mechanism potentially has beneficial effect in patients with high bone turnover, however the exact effect is still unknown in patients with low bone turnover. RANKL, the Receptor Activator of Nuclear factor Kappa-B Ligand
